# Early Aptian marine incursions in the interior of northeastern Brazil following the Gondwana breakup

**DOI:** 10.1038/s41598-023-32967-w

**Published:** 2023-04-25

**Authors:** Gerson Fauth, Henrique Parisi Kern, Jorge Villegas-Martín, Marcelo Augusto De Lira Mota, Marcos Antonio Batista dos Santos Filho, Amanda Santa Catharina, Lilian Maia Leandro, Fernanda Luft-Souza, Oscar Strohschoen, Andressa Nauter-Alves, Edna de Jesus Francisco Tungo, Mauro Daniel Rodrigues Bruno, Daiane Ceolin, Simone Baecker-Fauth, Marlone Heliara Hünnig Bom, Francisco Henrique de Oliveira Lima, Alessandra Santos, Mario Luis Assine

**Affiliations:** 1 Instituto Tecnológico de Paleoceanografia e Mudanças Climáticas (itt Oceaneon), Unisinos University, Avenida Unisinos, 950-Cristo Rei, São Leopoldo, RS 93022-750 Brazil; 2 Programa de Pós-Graduação em Geologia, Unisinos University, Avenida Unisinos, 950-Cristo Rei, São Leopoldo, RS 93022-750 Brazil; 3 Department of Geology, São Paulo State University-UNESP, Rio Claro, SP Brazil; 4 Petrobras, Research Center (CENPES), Av. Horácio Macedo, 950, Cidade Universitária, Ilha do Fundão, Rio de Janeiro, RJ 21941-915 Brazil

**Keywords:** Palaeoecology, Palaeontology, Stratigraphy

## Abstract

This study reports a set of primeval marine incursions identified in two drill cores, 1PS-06-CE, and 1PS-10-CE, which recovered the Barbalha Formation, Araripe Basin, Brazil. Based on a multi-proxy approach involving stratigraphy, microbiofacies, ichnofossils, and microfossils, three short-lived marine incursions were identified, designated Araripe Marine Incursions (AMI) 1–3. AMI-1 and AMI-2, which occur within the shales of the Batateira Beds (lower part of the Barbalha Formation), were identified by the occurrence of benthonic foraminifera, calcareous nannofossils, dinocysts, and a mass mortality event of non-marine ostracods. AMI-3 was recognized in the upper part of the Barbalha Formation, based on the occurrence of ichnofossils and planktonic foraminifera. The observation of the planktonic foraminifera genus *Leupoldina* for the first time in the basin indicates early Aptian/early late Aptian age for these deposits, and the first opportunity of correlation with global foraminifera biozonation. Our findings have implications for the breakup of the Gondwana Supercontinent, as these incursions represent the earliest marine-derived flooding events in the inland basins of northeastern Brazil.

## Introduction

The Aptian (126.3 to 113.1 Ma^1^) is an important stage of the Early Cretaceous, characterized by significant paleogeographic and paleoceanographic events around the world. Oceanic gateways gradually opened during this stage, and rising sea levels allowed the dispersion of marine biota (e.g.^2,3^). During the Early Cretaceous, the Gondwana Supercontinent broke up into the South American and African continents, leading to the establishment of the South Atlantic Ocean (e.g.^4^).

The initial evolution of the South Atlantic Ocean has been widely debated in terms of relative motion and timing. Reversal-related magnetic anomalies indicate that onset of the opening occurred around Anomaly M13 (134 Ma^5^) in the Austral Atlantic Ocean, but the Cretaceous Normal Polarity Superchron (C34n; 121 to 83.65 Ma^6^) complicates age determination for its subsequent spread northwards, between the Barremian and Aptian interval.

Identification of the source and pathways taken by the first marine flooding episodes from the South Atlantic Ocean into the Brazilian intracratonic sedimentary basins is further complicated by conflicting results based on paleontological and sedimentary proxies (e.g.^7–9^). Paleontological data supports a clear connection with Tethyan sea waters (e.g.^7^), while a combination of stratigraphy and geometry of the deposits establishes a transgression path from south to north and further inland^8,10,11^.

The geological record of northeastern Brazilian marginal and interior basins is fundamental for obtaining temporal and geographical constraints for the first marine incursions in the region, including their provenance and relationship with the relative motions between the incipient African and South American plates. A combination of paleontological, sedimentological, stratigraphic, and geochemical approaches is essential to obtain a clearer image of these ingressions and the relationship between the Tethys and the proto-South Atlantic waters, as well as the development of a more accurate paleogeographical model for this time interval. The recognition of marine incursions within these basins provides essential information to help decipher the pathways of this major geological event.

In this study, we provide a multi-proxy analysis of two boreholes, 1PS-06-CE and 1PS-10-CE, from the eastern portion of the Araripe Basin, Northeast Brazil. The origin of this large interior basin is related to the breakup of the Gondwana^11–14^, and late Aptian–early Albian marine incursions have already been identified (e.g.^7,8,15–20^). Our work yielded new ichnological, micropaleontological, and microbiofaciological data for the Barbalha Formation, the lowermost stratigraphic unit of the post-rift sequence of the basin and allowed identification of the primeval marine incursions in northeastern Brazil.

## Geological setting

The Araripe Basin is a large inland basin in northeastern Brazil, associated with the opening of the South Atlantic Ocean^10^. Its development, along several other rift basins in Brazil, was triggered by the breakup of the Gondwana Supercontinent during the Cretaceous^11–14,21^, and its deposits lay atop Precambrian terrains (Piancó-Alto Brígida and Granjeiro), in the transversal domain of the Borborema Province, to the south of the Patos Shear Zone.

The Santana Group (sensu Neumann and Assine^22^) is the stratigraphic record of the local Alagoas Stage (Aptian/lower Albian), equivalent to the post-rift I megasequence^11^. The precise age of the deposits of this group is the subject of considerable debate (e.g.^7,15,18,19,23–25^). Though typically assigned to the Aptian, several studies place the upper part of this unit (Romualdo Formation) in the Aptian–Albian interval (e.g.^15,23^).

**Figure 1:**
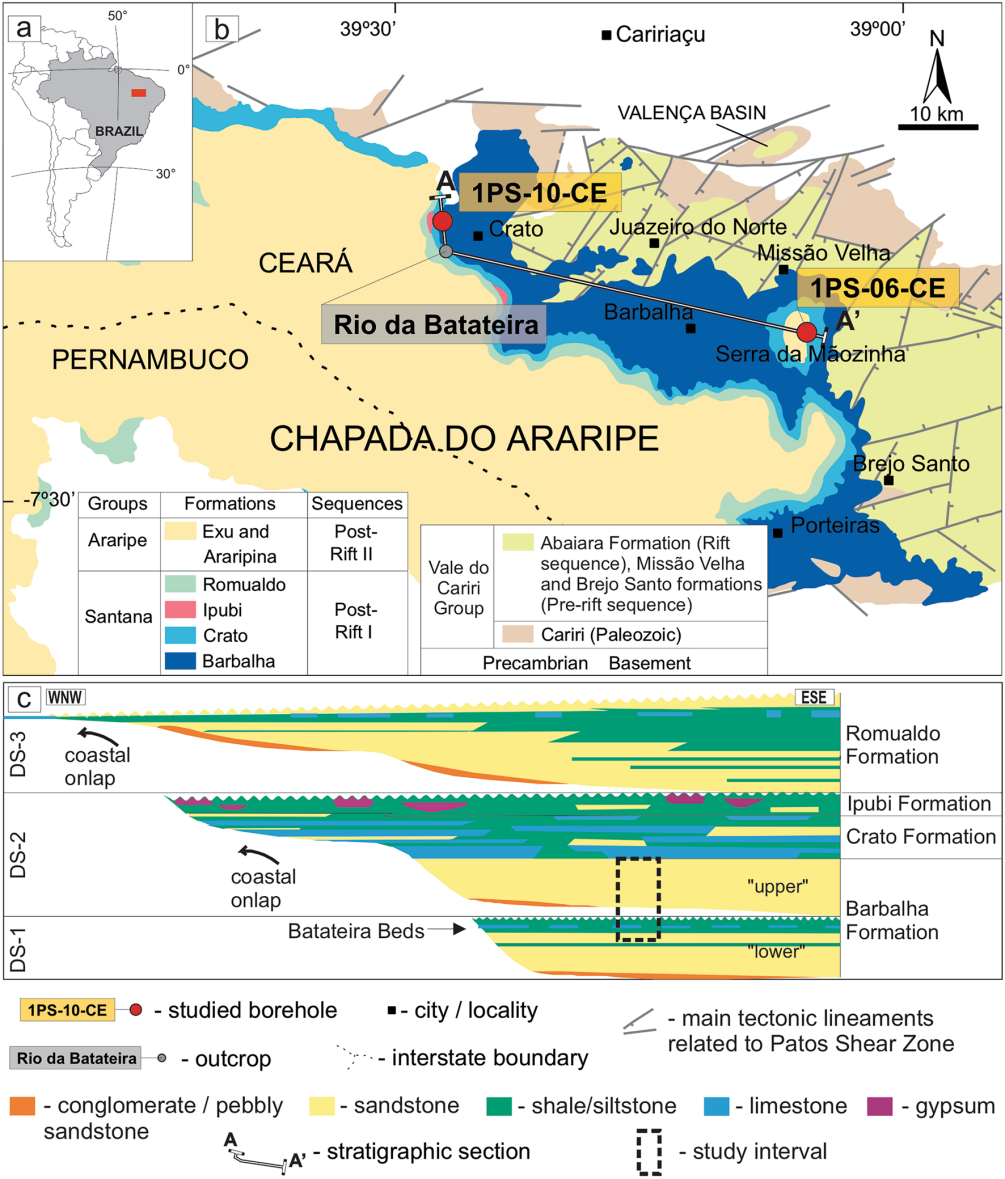
Geographic location and stratigraphic position of the studied area. (**a**) Map of South America and studied area (red polygon). (**b**) Simplified geological map of the Araripe Basin with location of the boreholes used in this study, modified from Assine et al.^8^. (**c**) Stratigraphic framework of the Alagoas Post-rift Stage in the Araripe Basin, composed of three depositional sequences bounded by disconformities. *DS-1* depositional sequence 1, *DS-2* depositional sequence 2, *DS-3* depositional sequence 3, modified from Assine et al.^8^. Graphic art made with CorelDRAW Graphics Suite × 6, version 16.0 (https://www.coreldraw.com/).

The sedimentary succession of the Santana Group is represented by the Barbalha, Crato, Ipubi, and Romualdo formations (Fig. 1), from base to top, with mixed carbonate-siliciclastic deposits^8,11^. The Barbalha Formation, focus of this study, is divided into two parts^8^. The lower one is essentially siliciclastic, passing from a fluvial to a lacustrine continental environment in a transgressive pattern that culminates in the Batateira Beds, a laterally extensive layer of mudstones and marls that are an important stratigraphic marker for the basin^8^. The type of preservation of the fossils in these beds corroborates an interpretation of continental environment setting, with dysoxic/anoxic intervals^11,12,26–28^. The upper part of the Barbalha Formation is composed of a siliciclastic succession of sandstones and mudstones with a fining upward pattern, that ends with the deposition of the laminated limestones of the Crato Formation reflecting a change from fluvial to hypersaline lacustrine environments (e.g.^29^).

Widespread marine ingressions have been reported in the Romualdo Formation, during the late Aptian of the basin^8,30^. Its deposits of black shales with fossil-bearing carbonate concretions (e.g.^16,31^) are famous for their well-preserved fossils, and include continental (palynomorphs and ostracods^15^) and marine groups (dinoflagellates^18^, benthonic and planktonic foraminifera^19,20^, marine ostracods^20^, and calcareous nannofossils^20^). The distribution of these marine organisms, many related to the Tethyan Realm, suggests short marine incursions from the Central Atlantic Ocean (e.g.^7,20,32,33^), which could have occurred due to sufficient tectonic subsidence rates, accompanied by sea level rise episodes^9,34^ at different times during the Early Cretaceous.

## Results

### Sedimentology and ichnology

**Figure 2:**
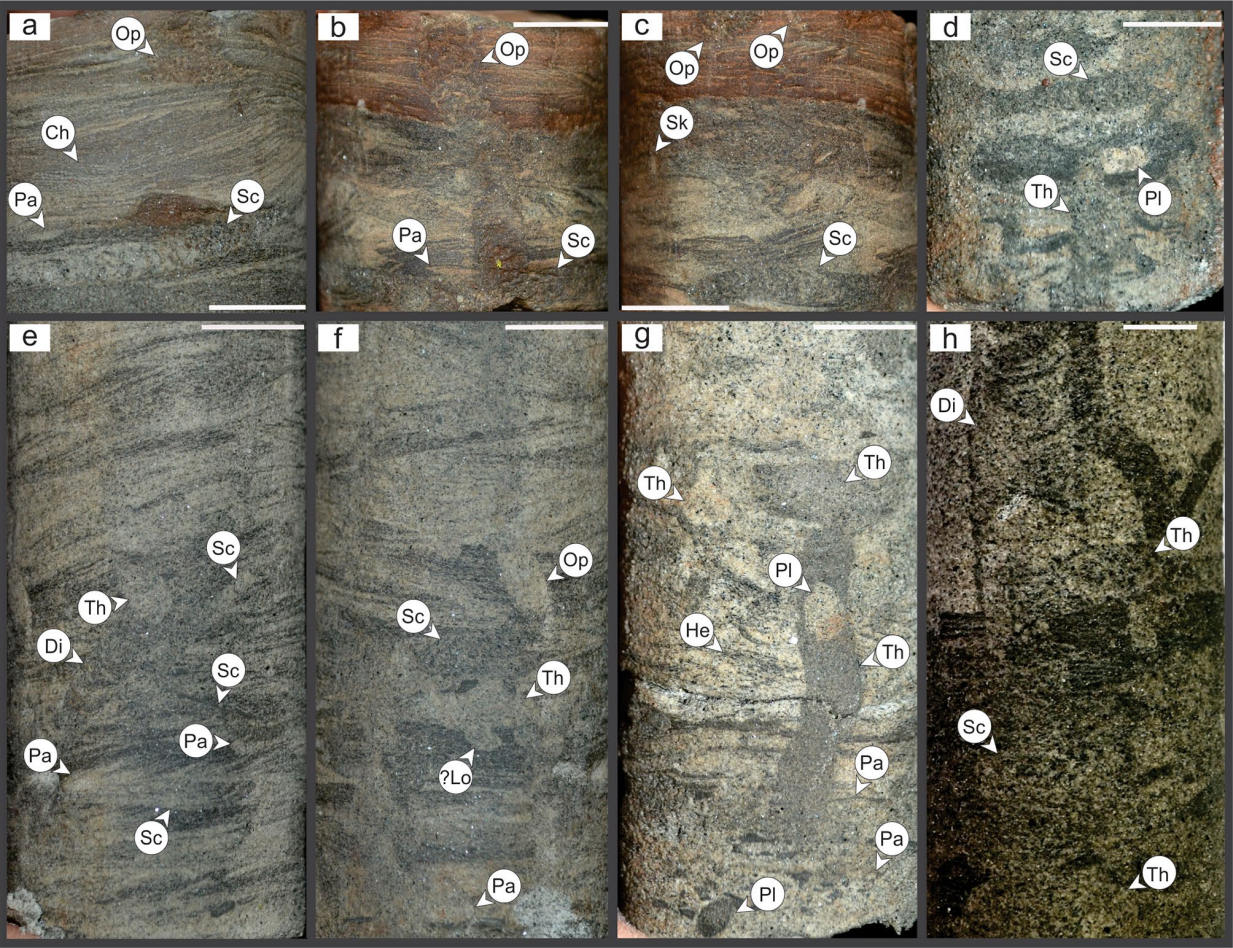
Trace fossils identified in the ichnofabrics of the studied section from Barbalha Formation (1PS-06-CE and 1PS-10-CE boreholes). (**a–c**) *Ophiomorpha* (Op), *Scolicia* (Sc), *Palaeophycus* (Pa), and *Skolithos* (Sk) (1PS-06-CE, 66.40 m). (**d**) *Scolicia* (Sc), *Thalassinoides* (Th), and *Planolites* (PL) (1PS-06-CE, 57.00 m). (**e,f**) *Scolicia* (Sc), *Thalassinoides* (Th), *Diplocraterion* (Di), *Palaeophycus* (Pa), *Ophiomorpha* (Op), and *Lockeia* (Lo) (1PS-06-CE, 68.50 m). (**g**) *Thalassinoides* (Th), *Helminthopsis* (He), *Planolites* (Pl), and *Palaeophycus* (Pa) (1PS-06-CE, 54.50 m). (**h**) *Diplocraterion* (Di), *Thalassinoides* (Th), and *Scolicia* (Sc) (1PS-10-CE, 33.50 m). Scale bars: 10 mm.

**Figure 3:**
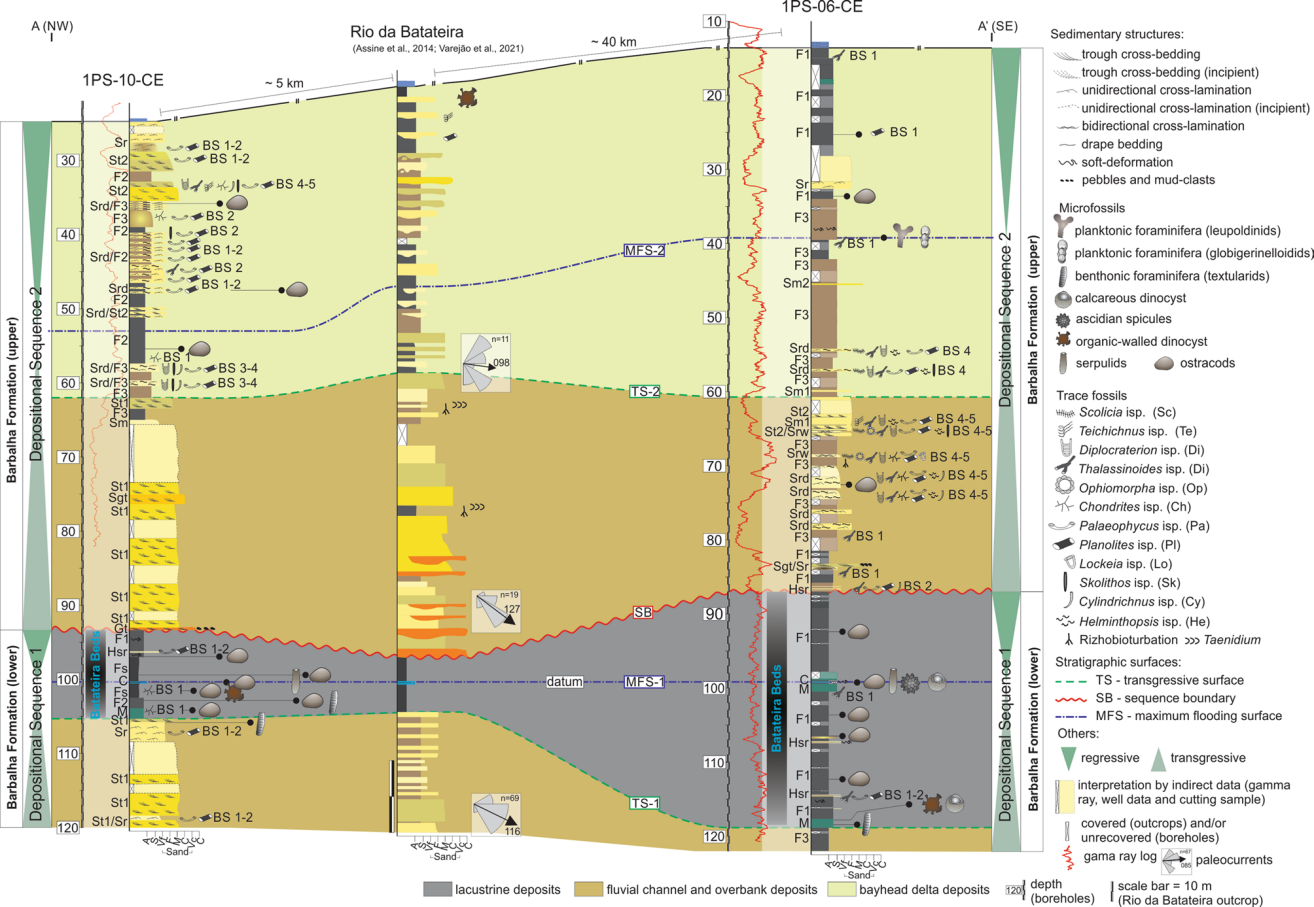
NW–SE stratigraphic section (location in Fig. 1b). Rio da Batateira sedimentary section adapted from Assine et al.^8^ and Varejão et al.^34^. Graphic art made with CorelDRAW Graphics Suite × 6 version, 16.0 (https://www.coreldraw.com/).

We recognized 16 sedimentary facies in the Barbalha Formation (Table 1). The lower part of this unit comprises sandstones and rare muddy deposits, overlain by laminites (Batateira Beds), with low ichnodiversity and low rates on the bioturbation scale (BS). The upper part is characterized by sandstone cosets (locally bioturbated), occasionally intercalated with fine-grained deposits (Fig. 2), overlaid by dominantly muddy deposits with interbedded sandstones (Fig. 3).

**Table 1:** Sedimentary lithofacies identified in the Barbalha Formation (upper and lower parts).

Lithofacies code	Description	^a^Bioturbation/bioturbation scale (BS)	Barbalha Formation
Fs	Dark gray clayey shale with moderate fissility. Sparse occurrence of bivalve shell impressions (~ 2 mm)	Ch/BS-1; Th/BS 2	Lower
F1	Medium gray to gray-green silty shale, massive to laminated, with low fissility, micaceous, occasionally interbedded with laminae of very fine-grained sandstone forming centimeter-thick heterolithic levels. Fluidized levels occur. Local abundance of plant debris	Pl/BS 1; Th/ BS 1; Th-Pl/BS 1	Lower
F2	Medium gray to cream-gray siltstone, with yellowish and reddish spots, micaceous and laminated. Rarely interbedded with discrete laminae of very fine-grained sandstone	Ch/BS-1; Pl/BS 1	Lower and upper
F3	Reddish-gray to light reddish-brown siltstone to claystone with greenish-gray spots, micaceous, massive to laminated. Thin levels of silty sandstone and fluidized levels occur. Intervals with blocky texture and slickenside structure also occur (paleosol)	Rhizobioturbation	Upper
M	Light gray marl, massive to laminated. Moderate to strong effervescence at 10% HCl	Th/BS 1–2; Ch-Pa/BS 1–2	Lower and upper
Hsr	Heterolith composed of centimeter-thick laminae of very fine-grained sandstone and dark gray siltstone. The sandstone has unidirectional cross-lamination, occasionally with claystone flaser bedding. Levels with moderate bioturbation and with fluidization occur	Pa-Pl-Th/BS 1–2; Pa-Pl/BS 1–2; Th/BS 2–3	Lower and upper
C	Laminite of millimetric- to centimetric-thick fine-grained limestone with parallel and/or crenulate lamination, interlayered with millimetric to centimetric beds of dark shale. Oval-shaped carbonate nodules and spherical carbonate concretions (pisoliths?) present. Convoluted aspect in 1PS-06-CE borehole. Petrographic analyzes reveal that the limestone is composed of ostracod wackestone and packstone, and microbially-induced sedimentary structures (MISS)	Pa-Ch/BS 1–2	Lower
Sr	Very fine- to fine-grained, cream-yellow to reddish sandstone, composed of sub-rounded to rounded quartz and feldspar grains, and lamellae of mica. Unidirectional cross-lamination occurs	Pa-Pl/BS 1–2/Pa-Pl/BS 1–2/Th/BS 1	Lower and upper
Srw	Very fine-grained gray sandstone, composed of sub-rounded quartz grains, white mica lamellae and gray claystone intraclasts. Bidirectional cross lamination present, with heavy minerals in the lamination planes	Sc-Th-Di-Pa-Pl-Ch-?Lo/BS 4–5; Op-Th-Pa-Pl-He-Di-?Sk/BS 4–5. Figure 2a–c; f	Upper
Srd	Very fine- to fine-grained gray sandstone, composed of sub-rounded quartz grains, opaque minerals, white mica lamellae and gray claystone rip-up clasts. Unidirectional cross-lamination with drape bedding present	Th/BS 1; Di-Pa-Pl-Th-Ch-Cy-He/BS 4–5; Di-Pa-Pl-?Sc-He-Th/BS 4; Th-Sc-Pa-Pl-Di-He/BS 4; Sk-Di-Pa-Pl-Cy-Bi/BS 3–4; Th-Pa-Pl/BS 2; Th-Pa-Pl/BS 2; Pa-Pl/BS 1–2; Pa-Pl-Cy/BS 1–2. Figure 2d,e; g	Upper
Sm1	Very fine-grained sandstone, gray and massive	Bioturbated aspect when associated with St2 facies	Upper
Sm2	Medium- to coarse-grained gray-white sandstone, massive, composed of sub-rounded quartz and feldspar grains, cemented by CaCO_3_	–	Upper
St1	Fine- to very coarse -grained brownish-yellow sandstone, composed of sub-rounded quartz and feldspar grains and mica lamellae. Trough cross bedding present (10–30 cm thick sets)	–	Lower and upper
St2	Fine- to medium-grained gray sandstone, with small- to medium-scale trough cross bedding. Composed of rounded to sub-rounded grains of quartz and feldspar, opaque minerals, and lamellae of mica. Heavy minerals in the bedding planes and levels with claystone intraclasts may occur. In the 1PS-06-CE well massive levels associated with intense bioturbation (Sm1) occur	Pa-Pl/BS 1–2; Di-Th-Cy-Pa-Pl-Te-Ch-?Sk/BS 4–5; Th-Pa-Pl-He-Di/BS 4–5. Figure 2h	Upper
Sgt	Pebbly sandstone with medium-grained quartz-feldspar matrix, light gray, with small-scale trough cross bedding (10 cm thick). Granules and small pebbles of quartz and altered feldspar (kaolin) occur	–	Upper
Gt	Small- to medium-size cobble conglomerate with fine- to coarse-grained sandstone matrix, composed of angular and sub-rounded quartz and feldspar grains, and mica lamellae. Small-scale trough cross bedding (10 cm thick). The pebbles are sub-rounded to sub-angular, small to medium sized, of quartz, feldspar, and lithic fragments (granitoids)	–	Upper

^a^Ichnofossils: Ch, *Chondrites* isp.; Th, *Thalassinoides* isp.; Pl, *Planolites* isp.; Pa, *Palaeophycus* isp.; Di, *Diplocraterion* isp.; Cy, *Cylindrichnus* isp.; He, *Helminthopsis* isp.; Sc, *Scolicia* isp.; Op, *Ophiomorpha* isp.; Sk, *Skolithos* isp.; Te, *Teichichnus* isp.; Lo, *Lockeia* isp; Bi, bioturbation indeterminated.

### Ostracods

**Figure 4:**
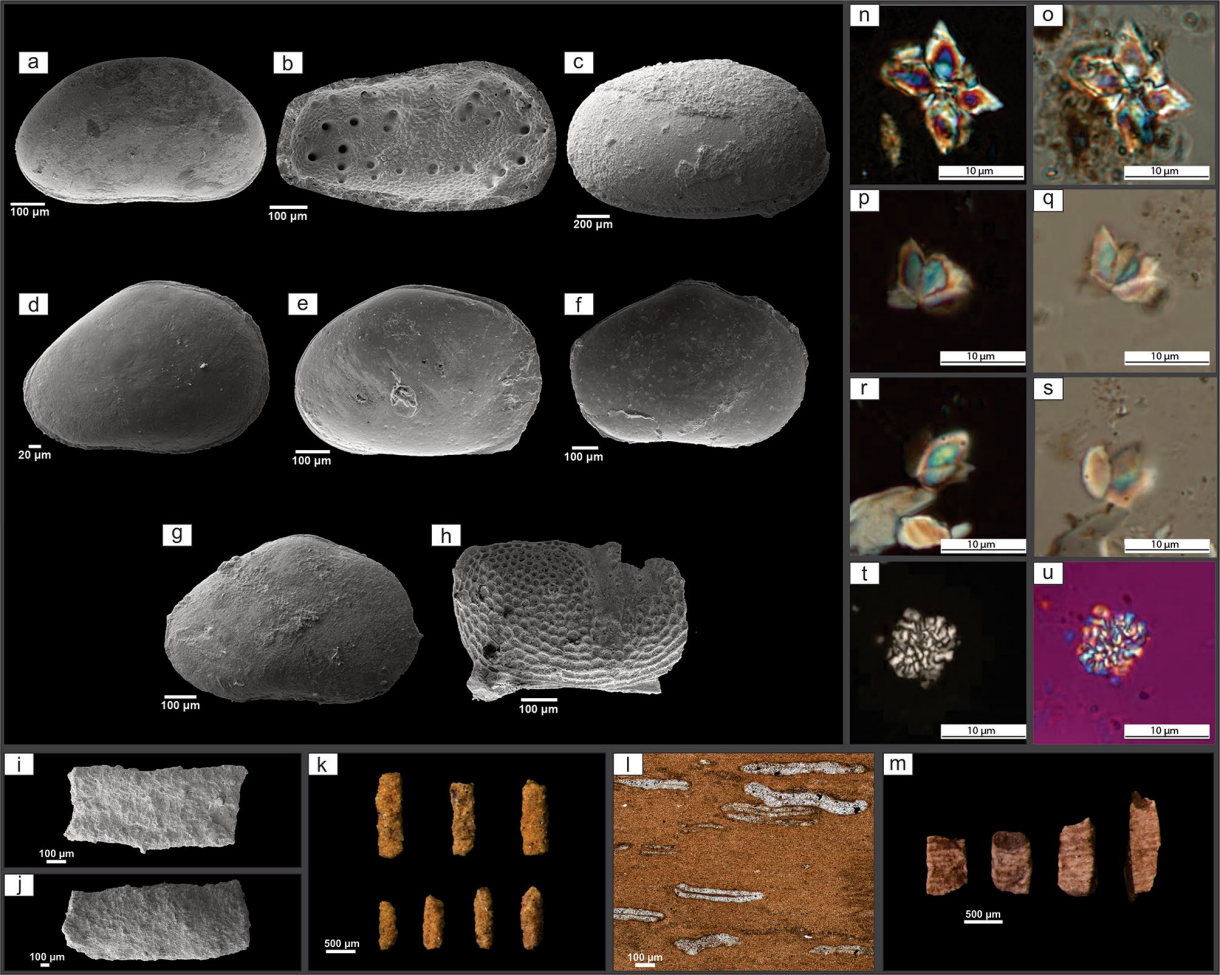
Plate illustrating key microfossil species recovered from boreholes 1PS-06-CE and 1PS-10-CE. Ostracods: (**a**) *Candonopsis? alagoensis* (right lateral view; 1PS-06-CE, 99.30 m); (**b**) *Cypridea araripensis* (mold; 1PS-10-CE, 51.20 m); (**c**) *Brasacypris subovatum* (right lateral view; 1PS-10-CE, 95.76 m); (**d**) *Pattersoncypris alta* (right lateral view; 1PS-06-CE, 99.30 m); (**e**) *Pattersoncypris salitrensis* (right lateral view; 1PS-06-CE, 99.30 m); (**f**) *Pattersoncypris angulata*^17^ (1PS-06-CE, 99.30 m); (**g**) *Pattersoncypris micropappilosa* (right lateral view; 1PS-06-CE, 99.30 m); (**h**) *Theriosynoecum silvai* (right valve, 1PS-10-CE, 95.76 m). Foraminifera: (**i–m**) *Bathysiphon* sp. ((**i,j**) 1PS-06-CE, 117.90 m; (**k–m**) 1PS-10-CE, 103.69 m, and 102.90 m). Calcareous nannofossils: (**n–s**) Ascidian spicule (1PS-06-CE, 99.30 m). (**t–u**) *Thoracosphaera* spp. (1PS-06-CE, 114.70 m).

Eight species belonging to three genera of non-marine ostracods were identified in borehole 1PS-06-CE (Fig. 4a–h), with moderate to good preservation. Recovered species include *Candonopsis*? *alagoensis*, *Candona*? sp., *Pattersoncypris alta*, *Pattersoncypris micropapillosa*, *Pattersoncypris salitrensis, Pattersoncypris angulata, Pattersoncypris* sp. 1, and *Pattersoncypris* sp. 2.

Ostracod abundance varies throughout this borehole. Between depths 112.30 and 92.40 m, which encompasses the Batateira Beds, there is a remarkably high abundance of well-preserved and articulated specimens (particularly at depths 99.80 and 99.30 m, with over 1000 individuals in each sample), including many younger ontogenetic stages. Between depths of 91.45 and 13.60 m, abundance and diversity of the ostracod fauna decreases considerably.

Comparatively, abundance and diversity are lower in borehole 1PS-10-CE, and fossil preservation is moderate to poor. The samples with the highest abundance are from the laminites of the Batateira Beds (101.54 m). Six species belonging to six genera of non-marine ostracods were recovered (Fig. 4a–h): *Candonopsis*? *alagoensis*, *Cypridea araripensis*?, *Brasacypris subovatum, Pattersoncypris* sp. 3, *Theriosynoecum silvai*, as well as one Gen. et sp. indet. ostracod. Ostracod distribution can be observed in Fig. 3.

### Benthonic foraminifera

Abundant, monofaunistic associations of agglutinated (textularids) benthonic foraminifera, classified as *Bathysiphon* sp., were found near the base of the Batateira Beds in both boreholes (117.90 m in 1PS-06-CE and 105.90, 103.69, 103.20, and 102.90 m in 1PS-10-CE) (Fig. 4i–m). Specimens are well-preserved but usually fragmented. Their distribution can be observed in Fig. 3.

### Calcareous nannofossils

Calcareous nannofossils were recovered only in borehole 1PS-06-CE, in the fine-grained facies of the Batateira Beds (Fig. 4n–u). We recorded poorly- to well-preserved ascidian spicules at 112.30 m and five specimens of calcareous dinocyst fragments (*Thoracosphaera* spp.) at 114.70 m.

We also observed 24 specimens of ascidian spicules at 99.30 m, the most abundant calcareous nannofossil assemblage of the borehole, and which are secreted by small (< 0.1 mm) soft-bodied tunicates that live in shallow water environments. They show diverse morphologies (rounded, spheric, and radial) as well as dissolution/overgrowth preservation effects. Four specimens of *Thoracosphaera* spp. found at 99.30 m have morphologies marked by fragments with large inner element sizes and low preservation. Calcareous nannofossil distribution can be observed in Fig. 3.

### Microbiofacies

**Figure 5:**
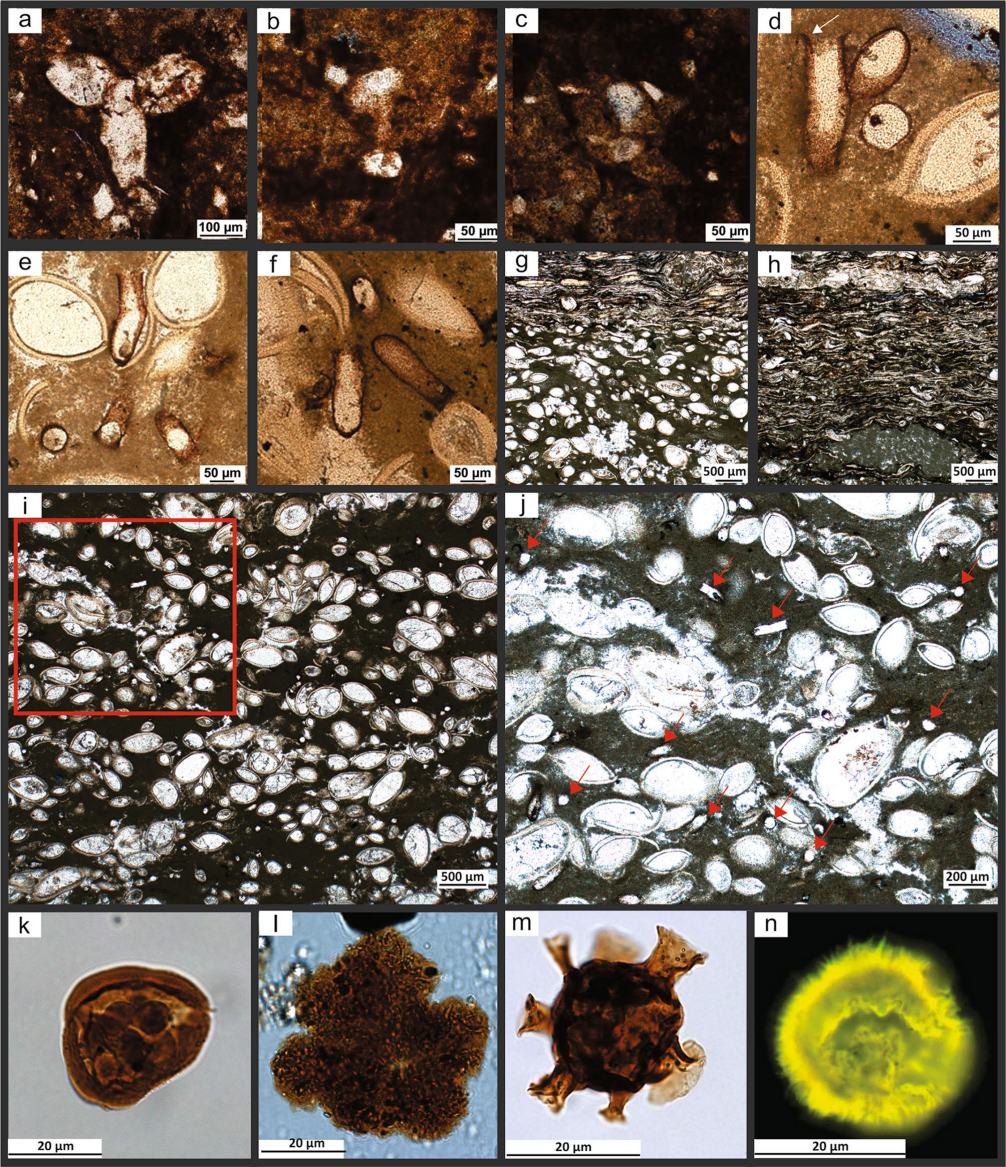
Key microfossil species recovered (1PS-06-CE and 1PS-10-CE boreholes): (**a**) *Leupoldina *sp. (1PS-06-CE, 39.20 m); (**b**) *Globigerinelloides *cf.* barri* (1PS-06-CE, 39.20 m); (**c**) *Globigerinelloides *cf.* ferreolensis* (1PS-06-CE, 39.20 m); (**d**) Serpulids (collar = white arrows) (1PS-10-CE, 100.45 m); (**e,f**) Serpulids (1PS-06-CE, 99.30 m); (**g,h**) Wackestone and packstone with ostracods (1PS-06-CE, 99.30 m); (**i**) Wackestone with ostracods and serpulids (red frame corresponds to image “(**j**)”) (1PS-06-CE, 99.30 m); (**j**) Detail view of wackestone with ostracod shells and serpulid tubes (red arrows) (1PS-06-CE, 99.30 m); (**k**) *Sergipea variverrucata* (1PS-10-CE, 104.2 m); (**l**) *Botryococcus* sp. (1PS-10-CE, 104.2 m); (**m**) *Oligosphaeridium* sp. (1PS-06-CE, 115.8 m); and (**n**) *Cometodinium* sp. (1PS-10-CE, 101.5 m; fluorescence mode).

Based on thin sections of boreholes 1PS-06-CE and 1PS-10-CE, we observed three horizons with marine microfossils (textularids, serpulids, and planktonic foraminifera) in the Barbalha Formation (Figs. 4l and 5a–j). The first horizon is a mudstone with abundant textularids, *Bathysiphon* sp. (at 103.69 m in borehole 1PS-10-CE). The second horizon consists of wackestone and packstone laminites (99.30 m in borehole 1PS-06-CE, and 100.63 m and 100.38 m in borehole 1PS-10-CE), with abundant ostracods, serpulid tubes (Fig. 5d–j), microbial pellets, organic matter, and calcareous concretions. Two distinct ostracod deposits were preserved in these laminites: a packstone with disarticulated (reworked) and compressed ostracod valves (Fig. 5i–j), and a wackestone with articulated, adult- to juvenile-sized ostracod carapaces, associated with serpulid tubes in an organic-rich matrix (Fig. 5j), interpreted as in situ. The serpulids observed are 50–250 µm long tubes with thin microcrystalline calcite walls (dark color in thin sections) and usually well-developed collars (Fig. 5d). Transverse sections are circular to ovoid, well-preserved, with partial dissolution and locally compressed. They are most abundant on the organic muddy microbial matrix among ostracod valves (Fig. 5g–j) but are absent in the ostracod packstone.

The third horizon is represented by the reddish-brown, cemented mudstone of the F3 facies (39.20 m depth, borehole 1PS-06-CE). Planktonic foraminifera specimens are frequent (Fig. 5a–d) and include the genera *Leupoldina* and *Globigerinelloides*. Most specimens have undergone some degree of dissolution, and internal molds, partially dissolved tests, and ghosts are prevalent. Poor preservation hinders species-level identification for leupoldinids (described as *Leupoldina* sp.) and  globigerinelloidids (*Globigerinelloides* cf. *barri* and *G.* cf. *ferreolensis*) (Fig. 5a–c).

Genus *Leupoldina* is characterized by pseudo-planispiral coiling, radially elongate chambers, and branched disaxial outer chambers, which makes the last whorl asymmetrical. In thin sections, early chambers are globular, and final chambers may be elongated, ovoid to circular, depending on the orientation of the cut, commonly with paired projections arranged symmetrically on each side of a median plane. Paired projections (branching) of final two chambers are typical for most species of *Leupoldina* (Fig. 5a). *Globigerinelloides* specimens were identified by their typical planispiral biumbilicate coiling in equatorial sections.

### Organic-walled microfossils

Pollen grains and plant spores (e.g., *Classopollis classoides*, *Inaperturopollenites* spp., *Araucariacites australis* and *Cicatricosisporites* spp.) are responsible for the majority of palynomorph counts in samples from boreholes 1PS-06-CE and 1PS-10-CE, followed by freshwater algae (*Botryococcus* sp. and *Pediastrum* spp.), with punctual occurrences in the Batateira Beds of dinocysts, *Oligosphaeridium* sp. at 115.80 m on 1PS-06-CE, and *Cometodinium* sp. at 101.54 m on 1PS-10-CE (Fig. 5k–n).

## Discussion

### Biostratigraphic framework and age controls

The stratigraphic positions of the biomarkers discussed in this chapter are shown in the Supplementary Material (SM).

The absence of globally distributed marine microfossils in the Brazilian interior basins has historically prevented their correlation with global chronostratigraphic charts, therefore they have only been calibrated using local biozones so far. Here we report for the first time the occurrence of *Leupoldina* spp. in the Barbalha Formation, in thin sections (Fig. 5a).

**Figure 6:**
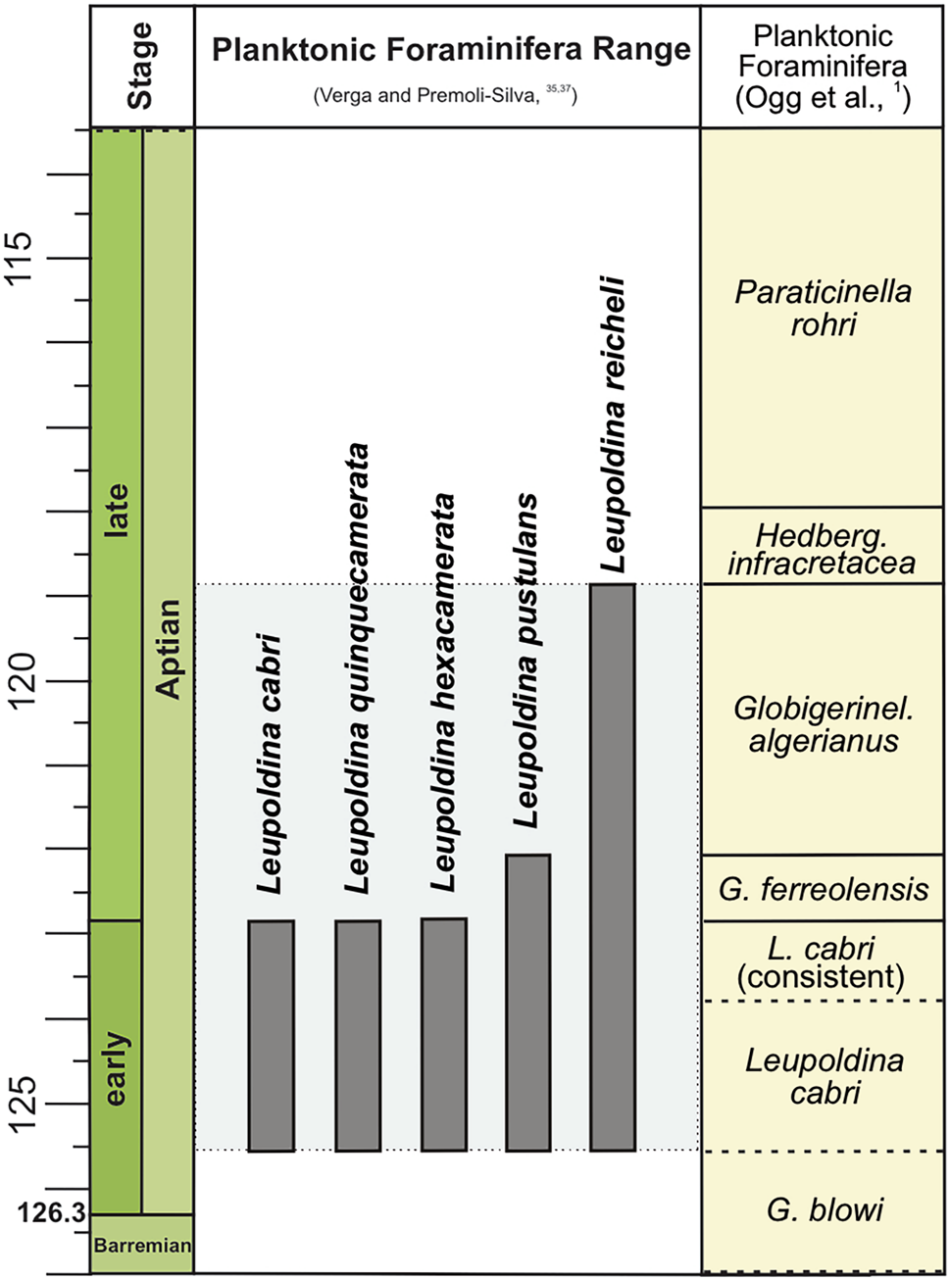
Planktonic foraminifera zonation scheme for the late Barremian–Aptian interval. Planktonic foraminifera range is taken from Verga and Premoli-Silva^35,37^ (adapted from Ogg et al.^1^). Graphic art made with CorelDRAW Graphics Suite × 6, version 16.0 (https://www.coreldraw.com/).

*Leupoldina* has numerous occurrences in the Tethyan region^35^, and is used in global foraminiferal biozonation schemes^36,37^ due to its distinct morphology from other planktonic foraminifera of this interval. Poor preservation and cut orientation limited species-level identification of *Leupoldina* specimens, but their occurrence, associated with *Globigerinelloides*, does allow us to assign biozones *L. cabri* to *G. algerianus*^36^. corresponding to the early Aptian/early late Aptian stage^1^ (Fig. 6). This outcome challenges the local biostratigraphic framework^15,38^ showing that the chronostratigraphic framework for the post-rift sequence in the Araripe Basin must be reviewed.

Concerning the local zonation schemes, the presence of the age-indicative ostracods *Pattersoncypris* spp. and *Theriosynoecum silvai* at boreholes 1PS-06-CE and 1PS-10-CE indicates local Alagoas Stage (Aptian–early Albian) for both sites (Zone 011; also called the *Pattersoncypris* and *Krommelbeincypris* Zone^39^).

Furthermore, the occurrence of age-indicative pollen grains belonging to the species *Sergipea variverrucata* (local Zone P-270^40^) constrains the studied intervals to a late Aptian age.

The lack of representatives of typical lower Aptian palynomorph-based biozones is probably due to paleoclimate exclusion^41^. We contend that paleoclimate dynamics might have controlled the local appearance and disappearance of plant species, which directly affected the palynostratigraphic records.

### Multi-proxy evidence of marine incursions

**Figure 7:**
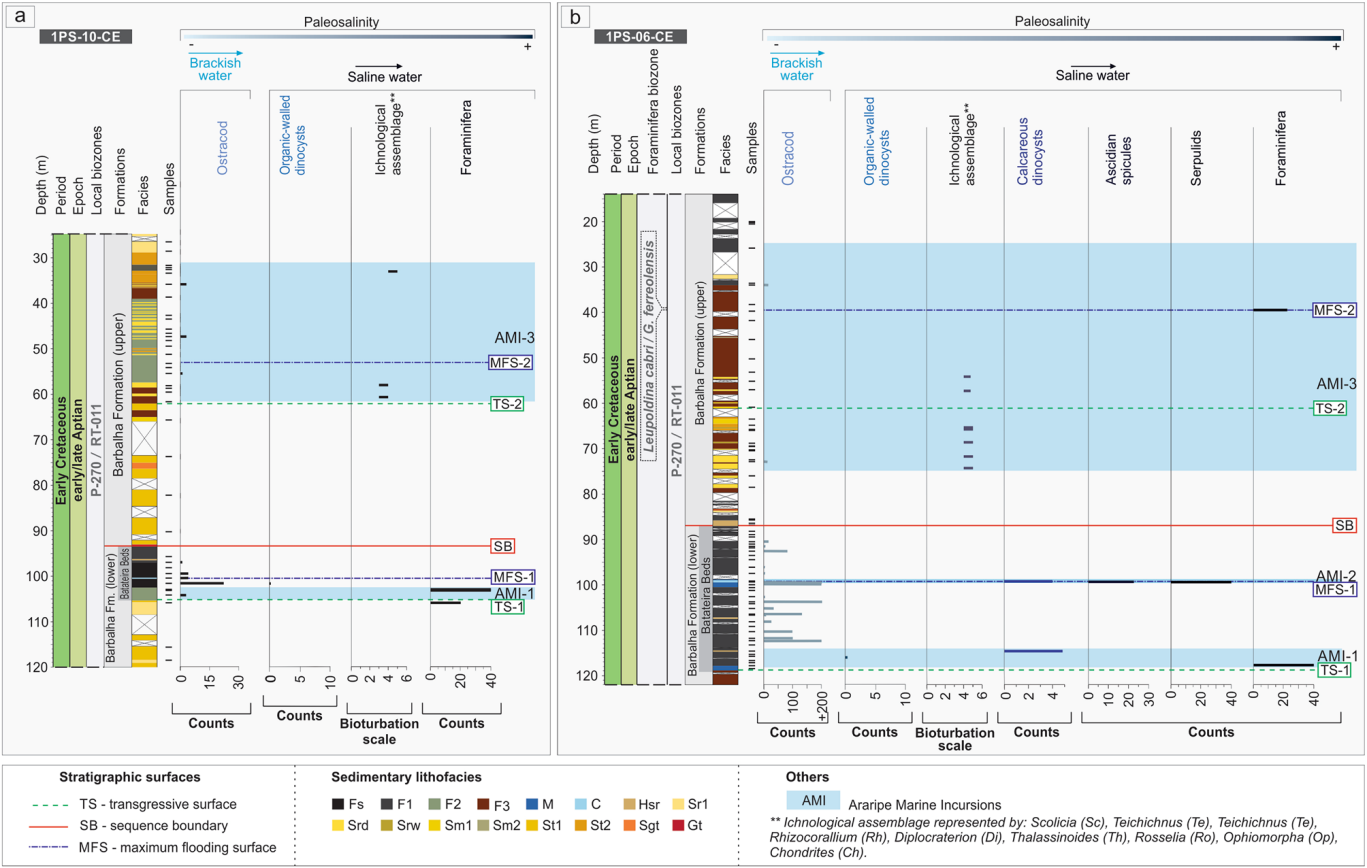
Plots of marine proxies’ analyses, with salinity indicators and identification of marine incursions in (**a**) 1PS-10-CE borehole, and (**b**) 1PS-06-CE borehole. Ostracod, palynomorph, and foraminifera biozones according to Poropat and Colin^39^, Regali and Silva Santos^40^, and Ogg et al.^1^, respectively. Graphic art made with CorelDRAW Graphics Suite × 6, version 16.0 (https://www.coreldraw.com/).

Three marine transgressive events, here named Araripe Marine Incursions (AMI), are clearly identifiable in the Barbalha Formation (Fig. 7). The Depositional Sequence 1 (sensu Assine et al.^8^) starts with very coarse- to coarse-grained sandstone facies (St2), superimposed by layers of fine- to very fine-grained sandstone with ripples (Sr) (Fig. 3; Table 1). Muddy levels (F6 and F1) are observed in lateral associations, sometimes interbedded with rippled sandstone (Sr), and as suggested previously, correspond to fluvial channels with laterally well-developed overbank^42^ (Fig. 3; Table 1).

Paleocurrent data reveal preferential flow towards SE^8,10,27,42^. The presence of agglutinating foraminifera in the sandstone deposits (1PS-10-CE) indicates brackish conditions possibly due to deltaic influence in this system.

The Batateira Beds^43^ overlie this fluvial system, with intercalating siliciclastic (F1 and F2) and heterolithic facies (Hsr). They have been largely classified as a lacustrine system (Fig. 3; Table 1) due to the occurrence of non-marine elements such as ostracods, fish, and continental palynomorphs (e.g.^11,44^). We have also observed an abundance of non-marine ostracods (*Pattersoncypris micropapillosa*, *P. salitrensis*, *P. angulata*, *Pattersoncypris* sp. 1, *Pattersoncypris* sp. 2, *Candonopsis*? *alagoensis,* and *Candona*? sp.) in these deposits (Fig. 4a–h). However, we present ichnological and micropaleontological data that supports input of marine waters in two distinct intervals, producing a salinity gradient between brackish to freshwater conditions in this lacustrine paleoenvironment (Fig. 7).

The first marine incursion (AMI-1) can be observed in both boreholes, in marls (facies M) just above transgressive surface TS-1 (Fig. 3 and 7). It is marked by abundant agglutinated foraminifera (*Bathysiphon* sp.) and the occurrence of calcareous and organic-walled dinocysts, marine elements that occur in association with an ichnofabric composed of *Planolites*, *Palaeophycus*, and *Thalassinoides*. The low ichnodiversity and occurrence restricted to facies F1 points to the establishment of opportunistic colonization due to the onset of brackish conditions^45,46^.

We identified the second marine incursion restricted to borehole 1PS-06-CE, AMI-2, in the middle part of the Batateira Beds, associated with its laminite deposits (C facies). It corresponds to a maximum flooding surface (MFS-1) (Figs. 3 and 7), recognized due to recovery of marine elements such as calcareous dinocysts (*Thoracoshpaera* spp.), calcareous nannofossils (ascidian spicules), and serpulid tubes. The thickness of these lacustrine deposits increases towards southeast, reaching 30 m in borehole 1PS-06-CE (Fig. 3). The presence of these marine elements, resting above the fluvial facies of both wells, reveals a generalized flooding event associated with an increase in the relative sea level.

Non-marine ostracod carapaces are particularly abundant in borehole 1PS-06-CE at the AMI-2 event (more than 1000 specimens recovered per sample at depths 99.80 and 99.30 m). The assemblage contains a large amount of well-preserved juvenile instars and adults, all with closed carapaces, indicating that they died at those life stages (Fig. 5g–j). Interestingly, carapaces of adult *Pattersoncypris*, a mixohaline genus, are smaller than usual^47^. All these features are likely result of development in a stressful environment and, eventually, a mass mortality event (e.g.^48,49^). Salinity changes in the depositional setting of the Batateira Beds could be responsible, as these ostracods did not tolerate marine conditions. This mortality event, associated with the presence of serpulids (Fig. 5i–j), foraminifera, calcareous nannofossils, and calcareous dinoflagellates, reinforces the hypothesis of establishment of fully marine conditions during this interval. Towards the top, the Batateira Beds record the reestablishment of lacustrine environments following a sea level drop.

The overlying coarse-grained facies record subaerial exposure, reactivation of the fluvial system, and the beginning of Depositional Sequence 2 (sensu Assine et al.^8^ and Scherer et al.^42^) generating a clear sequence boundary (SB; Fig. 3). Paleocurrent measurements from outcrops (e.g., Rio Batateira) show paleoflow direction mainly towards southeast^8,10,27,42,44^. Sandy deposits (Gt, Sgt, St1, and St2) of fluvial channels are predominant in proximal settings (borehole 1PS-10-CE), and facies F2 and F3 represent overbank deposits in an underdeveloped floodplain within the river system^27,34,42^. The blocky and slickensides structures, associated with rhizobioturbation in facies F3 also indicate eventual subaerial exposure.

Distally, in borehole 1PS-06-CE and above the SB, bioturbation is characterized by ichnofabrics composed of *Planolites*, *Palaeophycus*, *Thalassinoides*, and *Cylindrichnus*, as well as monospecific *Thalassinoides* ichnofabrics (Hsr, F1, and F3). The low ichnodiversity and low bioturbation scale values (BS = 1–2) reflect stressful conditions^45,46^ and are related to the colonization of softgrounds, possibly stressed by salinity fluctuations (e.g.^50^). They are an impoverished expression of the *Cruziana* ichnofacies due to the introduction of brackish conditions in the distal portions of the fluvial setting.

The beginning of AMI-3 is identified by the gradual increase of ichnodiversity and bioturbation scale values towards the top in distal settings. Bioturbation in facies Srd, Srw, and St2 is represented by ichnofabrics composed of *Diplocraterion*, *Ophiomorpha*, *Palaeophycus*, *Planolites*, *Thalassinoides*, *Chondrites*, *Helmintopsis*, *Scolicia*, *Lockeia,* and *Skolithos* (BS 1–5) (Table 1), indicating establishment of an impoverished expression of the mixed *Skolithos*-*Cruziana* ichnofacies (e.g.^50^). These ichnological characteristics might reflect stressful conditions caused by salinity changes. Moreover, the presence of *Scolicia* associated with the Srw lithofacies suggests that salinity was sufficient to sporadically support the establishment of a stenohaline fauna.

*Scolicia* has been mainly recorded in marine environments, from shoreface to deep-sea (e.g.^45^), and represents bioturbation by spatangoids echinoderms, which are truly marine organisms (e.g.^51^). The record of *Scolicia* in stressed substrates of distal, brackish, tidally-dominated, or -influenced channels is scarce (e.g.^52^). In addition, the higher ichnodiversity (marine elements) suggests increased marine conditions towards the top (Fig. 3).

These fluvial facies are overlain by muddy deposits (F3, F2, and F1) interbedded with sandstones (Sr, Srd, St2, and Sm), interpreted as bayhead delta deposits^34^ and suggesting flooding of the fluvial system (Fig. 3; Table 1). The ichnological assemblages and micropaleontological data support this interpretation of flooding in both boreholes.

In both boreholes, the bioturbation above the TS-2 is characterized by ichnofabrics composed of *Diplocraterion*, *Thalassinoides*, *Palaeophycus*, *Planolites*, *Chondrites*, *Cylindrichnus*, *Helminthopsis*, *Scolicia*, and *Skolithos*, with BS 3–4, and are restricted to the Srd lithofacies (Fig. 3). They represent the establishment of an impoverished expression of the mixed *Skolithos*-*Cruziana* ichnofacies (e.g.^50^) and continuous marine influence in the deposits at 1PS-06-CE.

The higher ichnodiversity, including *Scolicia* burrows in 1PS-06-CE, suggests that marine conditions were more stable in the southeastern area. In addition, the occurrence of planktonic foraminifera (*Leupoldina* sp. and *Globigerinelloides* spp.) in 1PS-06-CE, laterally associated with ichnological assemblages of the impoverished mixed *Skolithos*-*Cruziana* ichnofacies of 1PS-10-CE, shows that marine to brackish conditions followed the onset of the bayhead delta. The presence of *Leupoldina* sp. and *Globigerinelloides* spp. also points to the establishment of typical marine conditions in the upper Barbalha Formation, configuring the second maximum flooding surface (MFS-2) (Fig. 3).

## Conclusions

We dated the deposits of the Barbalha Formation for the first time based on the recovery of the planktonic foraminifera genera *Leupoldina* and *Globigerinelloides*. These genera mark the *L. cabri* to *G. algerianus* zones, which corresponds to the early Aptian/early late Aptian interval.

Three marine incursions (AMI-1 to AMI-3) were identified in the Barbalha Formation based on a multi-proxy analysis (micropaleontological, ichnological, and sedimentary analyses). The lower two occur in the Batateira Beds (lower Barbalha Formation), recording the primeval marine incursions into the Araripe Basin related to the breakup of the Gondwana, and the opening of the South Atlantic Ocean.

AMI-1, near the base of the Batateira Beds, is characterized by an abundance of the agglutinated foraminifera, and minor occurrence of organic-walled dinocysts and calcareous dinocysts.

AMI-2, in the laminite deposits of the Batateira Beds of borehole 1PS-06-CE, is characterized by calcareous dinocysts, ascidian spicules, microbial peloids, and abundant serpulid tubes. The mass mortality event of non-marine ostracods reinforces this marine incursion event.

AMI-3, in the upper part of the Barbalha Formation, is characterized by the occurrence of bioturbation mainly represented by *Scolicia*, *Diplocraterion*, *Thalassinoides*, *Ophiomorpha*, *Skolithos*, and *Chondrites*, as well as the presence of planktonic foraminifera *Leupoldina* spp. and *Globigerinelloides* spp.

These incursions are the oldest recorded so far related to the breakup of the Gondwana Supercontinent and the opening of the South Atlantic Ocean. Future studies with fossil records, geochemistry, and paleomagnetism will contribute to the characterization of these marine deposits, as well as the routes taken by these marine incursions.

## Materials and methods

### Sedimentary sections

We described and analyzed core samples taken from boreholes 1PS-06-CE and 1PS-10-CE, drilled by the Santana II Project^53^ in the eastern portion of the Araripe Basin, northeastern Brazil. Lithological, ichnological, microbiofaciological, and micropaleontological analyses were completed, preferentially done in the fine siliciclastic and limestone lithologies (shales, mudstones, siltstones, marls, and laminites), but also in some sandstone levels. For further details, we provide the full dataset of microfossils/microbiofacies in the supplementary materials (SM).

### Sedimentological, ichnological, and stratigraphic analysis

The characterization of the sedimentary facies of boreholes 1PS-06-CE and 1PS-10-CE followed the usual methods, with description of physical sedimentary structures and basic lithology, focusing on lithofacies and ichnology. Intervals with no recovery were interpreted based on the accompanying well drilling data, namely gamma-ray values (indirect data); a small portion was obtained from cut samples. For the purposes of stratigraphic correlation, the laminite deposits of the Batateira Beds, in the lower part of the Barbalha Formation, were chosen as our datum; specifically, the levels containing serpulids and the ostracods mass mortality event, which occur on both drill cores.

Direct macroscopic observation was used to describe trace fossils, following the main ichnotaxobases applicable to ichnofabrics: burrow limit, burrow infill, branching pattern, and presence/absence of spreiten. The guidelines used for ichnotaxonomy classification followed Bertling et al.^54^. In some cases, identification of ichnotaxa was hampered by the loss of ichnotaxonomical features, dark lithology, and/or core deformation, which erased this data, as well as the restrictions inherent to observations made using two-dimensional core surfaces.

The amount of bioturbation was quantified considering the average diameter of 4 cm for the cores and using the bioturbation scale (BS) proposed by Reineck^55^, which ranges from 0 (non-bioturbated) to 6 (completely bioturbated). Data from the Rio da Batateira outcrop^8,33^ was taken into consideration for the creation of the stratigraphic section, due to its geographical proximity and relevant correlation to the stratigraphic sequence.

### Calcareous microfossils

Samples were prepared for the recovery of both ostracods and foraminifera using the methodology developed for the rocks of the Romualdo Formation used by Bom et al.^49^, and consisted of the immersion of 20 g of sediment in 200 mL of deionized water with 3 mL of Extran for 24 h. The sediments were then washed through 250, 180, 63, and 45 µm sieves, and dried for 48 h in a lab oven at 40 ºC. After hand picking, we imaged the most representative specimens in an EVO/MA15 Zeiss scanning electron microscope (SEM).

No planktonic foraminifera species were recovered in washed samples, probably due to the strong cementation and poor preservation of the material in the sampled interval. Thus, their analysis was only possible through the thin sections made for microbiofacies studies.

All the studied material is currently stored in the micropaleontologic collection of the Museu de História Geológica do Rio Grande do Sul (MHGRS), Unisinos University, Brazil, under the curatorial numbers ULVG 13482 to ULVG 13491.

### Organic-walled microfossils

We processed approximately 40 g of each sediment sample for palynology analysis, following standard techniques^56^. Minimums of 200 palynomorphs were counted in each sample for the palynological method. Species scanning, identification and counting were carried out with a Zeiss Imager.A2 microscope, using bright field illumination and incident blue light (fluorescence mode) at 200× , 400× , and 1000× (oil) magnifications. Photomicrographs were taken using a Zeiss AxioCam MRc (Micropaleontology Reference Center) digital camera. Our palynological analysis recorded pollen grains, spores, dinocysts, and freshwater green algae. The slides are stored in the collection of the MHGRS, Unisinos University, Brazil, under the curatorial numbers ULVG 13595 to ULVG 13682.

### Calcareous nannofossils

Sample preparation for calcareous nannofossil analysis followed the decantation methodology described in Bown and Young^57^. Each sediment sample was fragmented in an agate mortar and placed in a Falcon tube with 40 mL of deionized water. The solution was stirred for 30 s and then set to decant for five minutes. The supernatant (approximately 0.2 mL) was then collected, poured onto a coverslip, and placed in a hotplate (60 °C) to dry. After dried, the coverslip was placed on a slide with Norland optical adhesive 61 and cured under UV light.

The slides were examined using a Zeiss Axio Imager.A2 microscope, at 1000× magnification. Data was processed using the software Zen 3.0 (blue edition) for micrometric measurements. The slides are stored in the collection of the MHGRS, Unisinos University, Brazil, under the curatorial numbers ULVG 13492 to ULVG 13594.

### Microbiofacies

Lithology and preservation degree throughout the cored sections controlled the sampling interval. Select intervals of alternated mudstone-packstone and shale-siltstone required higher sampling density, while coarser-grained intervals were strategically undersampled.

We used a ZEISS Axioscope 5 petrographic microscope for microfossil identification and lithologic analysis, with transmitted and polarized light, objectives of 2.5× , 5× , 10× , and 20× magnification, and an attached digital camera. Data was processed using the software Zen 3.0 (blue edition) for micrometric measurements.

Calcareous rocks were described according to the nomenclatures of Dunham^58^. The taxonomic classification of planktonic foraminifera used was based on Verga and Premoli-Silva^35,37^, as well as the online Mesozoic Planktonic Foraminifera database located at https://www.mikrotax.org (pforams@mikrotax^36^). Planktonic foraminifera zones were defined based on the biostratigraphic schemes of Ogg^1^. We defined six semi-quantitative categories representing relative abundance, based on the number of specimens of foraminifera counted: very abundant (> 40), abundant (20–40), common (10–20), few (5–10), rare (3–5), and very rare (1–3) (SM). The slides are stored in the collection of the MHGRS, Unisinos University, under the curatorial numbers ULVG 13685 to ULVG 13722.

## Supplementary Information

Supplementary Legends.

Supplementary Information.

## Data Availability

The authors confirm that the data supporting the findings of this study are available within the article and its supplementary materials.
