# Supplementary material for: Early Aptian marine incursions in the interior of northeastern Brazil following the Gondwana breakup
Source: Sci Rep. 2023 Apr 25;13:6728. doi: 10.1038/s41598-023-32967-w (PMC10130215; doi:10.1038/s41598-023-32967-w)
Supplement: Supplementary file 1 — Supplementary Legends. [file 41598_2023_32967_MOESM1_ESM.docx]

Supplementary Material 1. Distribution of calcareous microfossils and palynological content on boreholes 1PS-06-CE and 1PS-10-CE.
